# Isolation of active compounds from *Streptomyces sennicomposti* GMY01 and cytotoxic activity on breast cancer cells line

**DOI:** 10.1016/j.heliyon.2024.e24195

**Published:** 2024-01-05

**Authors:** Rifki Febriansah, Triana Hertiani, Jaka Widada, Muhammad Taher, Ema Damayanti, Mustofa Mustofa

**Affiliations:** a School of Pharmacy, Faculty of Medicine and Health Sciences, Universitas Muhammadiyah Yogyakarta, Indonesia; b Pharmacognosy and Phytochemistry Laboratory, Pharmaceutical Biology Department, Faculty of Pharmacy, Universitas Gadjah Mada, Yogyakarta, Indonesia 55281; c Department of Agricultural Microbiology, Faculty of Agriculture, Universitas Gadjah Mada, Yogyakarta, Indonesia 55281; d Department of Pharmaceutical Technology, Kulliyyah of Pharmacy, International Islamic University Malaysia, Malaysia; e Research Center for Food Technology and Processing, National Research and Innovation Agency (BRIN), Gunungkidul, Yogyakarta, Indonesia 55681; f Department of Pharmacology and Therapy, Faculty of Medicine, Public Health and Nursing, Universitas Gadjah Mada, Yogyakarta, Indonesia 55281

**Keywords:** *Streptomyces*, Compound isolation, Mannotriose, Anti-Breast cancer, *In vitro*

## Abstract

The occurrence of resistance to anticancer and the emergence of serious side effects due to chemotherapy is one of the main problems in cancer treatment, including breast cancer. The need for effective anticancer with a specific target is urgently required. *Streptomyces* are widely known as the potential producers of new anticancer molecules. Previously reported that the methanol extract of *Streptomyces sennicomposti* GMY01 isolated from Krakal Coast, Gunungkidul had very strong cytotoxic activity against MCF-7 and T47D breast cancer cells with IC_50_ values of 0.6 and 1.3 μg/mL, respectively. The following study aimed to isolate and identify active compounds of the *S. sennicomposti* GMY01 and evaluate its cytotoxic activity. The study was started by re-culturing and re-fermented optimization of *S. sennicomposti* GMY01 in a larger volume, then the bacteria were extracted using methanol following the bioassay-guided isolation of the extract obtained. The active compounds obtained were then structurally determined using UV/Vis spectroscopy, Fourier Transform-Infrared (FT-IR), Liquid Chromatography-Mass Spectroscopy (LC-MS), ^1^H NMR, and ^13^C NMR and analyzed for their cytotoxic activity using MTT assay on MCF-7 and normal Vero cells line. The results showed that the culture of the *S. sennicomposti* GMY01 using Starch Nitrate Broth (SNB) media yields the best results compared to other culture media. An active anticancer compound namely mannotriose was successfully isolated from the methanol extract with an IC_50_ value of 5.6 μg/mL and 687 μg/mL against the MCF-7 and Vero cells lines, respectively, indicating that this compound showed strong cytotoxic activity with high selectivity.

## 1 Introduction

Cancer is a cellular disease characterized by the disruption or failure of multiplication regulatory mechanisms and other homeostatic functions in multicellular organisms. This results in cells growing and developing uncontrollably, even then spreading and attacking other body tissues. Cancer is still a major health problem in the world due to its high morbidity and mortality. In 2020, it was estimated that 19.3 million new cases of cancer were reported with 10.0 million deaths [1]. Breast cancer is the second most deadly disease after lung cancer. In Indonesia in 2020, the cases of breast cancer were 65,858 with 22,430 deaths [2]. Based on the national health survey, the Yogyakarta Province, one of the 38 provinces of Indonesia, has more than twice the national cancer prevalence. In Yogyakarta, the majority of cases of breast cancer are diagnosed at the late stage, with Yogyakarta City having the highest proportion of diagnoses at stage 4. The trend of increasing breast cancer incidence is fastest in Yogyakarta City with an average percentage change per year of 18.77 % [3].

Several risk factors are associated with the development of breast cancer, namely cigarette smoke, alcohol consumption, age at first menstruation, age at first delivery, dietary fat, and family history [4]. Hormones also play an important role in the occurrence of breast cancer. Estradiol and progesterone in the normal menstrual cycle increase the risk of breast cancer. This occurs in breast cancer that has estrogen receptors, where about 50 % of breast cancer cases are estrogen-dependent cancers [5].

Although the molecular mechanisms that influence the risk of breast cancer and its progression have not been known with certainty, the activation of oncogenes due to genetic modification or the occurrence of epigenetic modifications can lead to the occurrence of breast cancer cell multiplication and migration [6]. Several oncogenes are reported to be involved in breast cancer carcinogenesis, including Ras, c-myc, epidermal growth factor receptor (EGFR, erb-B1), and erb-B2 (HER-2/neu) [7].

To find a new anticancer molecule, the screening was carried out through a combination of PKS I and NRPS gene analysis and TLC densitometry profile of *Actinomycetes* isolates from Krakal coast, Gunungkidul, Yogyakarta, Indonesia. Among 52 isolates tested, 14 isolates have cytotoxic activity, and one isolate showed the most potential activity against MCF-7 and T47D breast cancer cell lines with IC_50_ values of 0.6 μg/mL and 1.3 μg/mL, respectively [8]. *Actinomycetes* are gram-positive bacteria that have a high G + C DNA. *Actinomycetes* are one of the important sources of microbes in the development of biotechnology and medicine. Almost 70 % of natural antibiotics are produced by these bacteria. *Streptomyces* is the largest antibiotic-producing genus, although the role of other genera in drug discovery cannot be ruled out. The characteristic of most *Actinomycetes* is that the bacteria are mesophilic, i.e., bacteria will grow optimally at temperatures between 25 and 30 °C [9]. Furthermore, this also applies to the isolates of *Streptomyces* sp. GMY01.

Further identification showed that GMY01 isolate belongs to the *Streptomyces* genus and had the closest similarity with *Streptomyces sennicomposti* strain RCPT1-4T with an average nucleotide identity (ANI) and ANI based on BLAST+ (ANIb) values of 98.09 and 97.33 % (>95 %) [10]. In the previous study proved that the methanol fraction of the cultured isolates of the *Streptomyces* sp. GMY01 has potential cytotoxic activity against MCF-7 and T47D breast cancer cells with IC_50_ values of 0.8 and 127 μg/mL, respectively. The methanol extract was also shown to be non-toxic in normal NIH-3T3 cells with an IC_50_ value of 3707 μg/mL [8]. Genome mining analysis of *Streptomyces* sp. GMY01 showed that this isolate had 10 NRPS genes encoding various secondary metabolites from the previously known active compounds [11]. An *in vitro* bioassay of the GMY01 bioactive on cervical carcinoma of HeLa cell and lung carcinoma of HTB cells exhibited moderate activity with low toxicity on Vero cells as a normal cell. Metabolite profiling by LC-MS/MS analysis revealed that the active fraction of GMY01 contained carbohydrate-based compounds, C_17_H_29_NO_14_ (471.15880 Da) as a major compound (97.50 %) and mannotriose (C_18_H_32_O_16_; 504.16903 Da, 1.96 %) as a minor compound [10]. It is indicated that the isolate has the potential to be explored to discover new anticancer molecules.

This study was conducted to isolate active compounds from the methanol extract of *S. sennicomposti* GMY01 against the MCF-7 breast cancer cell line. Structure elucidation of the isolated active compound and cytotoxic test against the Vero normal cell line was also conducted. This study is still limited to *in vitro* study, the result of this study needs to be analyzed further by *in vivo* study to confirm the results.

## 2 Methods

### 2.1 Culture of *Streptomyces sennicomposti* GMY01 and preparation of extracts from culture fluids

*Streptomyces sennicomposti* strain GMY01 was isolated from a marine sediment sample collected from Krakal Beach (8°8′44″S 110°35′59″E), Gunungkidul Regency, Daerah Istimewa Yogyakarta Province, Indonesia [10]. *S. sennicomposti* GMY01 was deposited at the Indonesian Culture Collection, (World Data Center for Microorganisms, WDCM 769), Indonesian Institute of Sciences (LIPI) as InaCC A147 and NITE Biological Research Center (NBRC, WDCM 825) Japan with registration number NBRC 110111*. S. sennicomposti* GMY01 was maintained using International *Streptomyces* Project-2 (ISP-2) media and incubated at 30 °C. For initial fermentation, Tryptic Soy Broth (Difco, Sparks, MD) and Starch Nitrate Broth (SNB) were used as fermentation medium for 3–5 days. For final fermentation, GMY01 was cultured at 30 °C and agitated at 180 rpm for 3 days in a 250 mL Erlenmeyer flask containing 100 mL of TSB as the incolum. A total of 30 mL (10 % v/v) of 3 days inoculum culture was inoculated in 300 mL of Starch Nitrate Broth (SNB) media using a 500 mL Erlenmeyer tube, then incubated on a rotary shaker incubator at 200–250 rpm for 11 days at room temperature (25–30 °C). The SNB medium contained 0.5 g of NaCl, 1 g of KNO3, 0.5 g of K2HPO4, 0.5 g of MgSO4·7H2O, 0.01 g of FeSO4·7H2O, and 20 g of soluble starch in 1000 mL of distilled water [12]. The secondary metabolite was obtained by separating the cell biomass from the liquid culture using centrifugation at 3000 rpm for 15 min. The cell biomass obtained was then macerated using methanol for 1 h. After completion, it was filtered, and the supernatant was evaporated to obtain a solid extract for bioassay and active compound isolation.

### 2.2 Isolation of the active compound from the culture extract of *Streptomyces sennicomposti* GMY01 with bioassay-guided isolation method

The culture extract of *S. sennicomposti* GMY01 isolated its active compounds by bioassay-guided isolation method [13]. The culture of *S. sennicomposti* GMY01 was extracted using a maceration method with methanol as solvent. The methanol extract obtained from *S. sennicomposti* GMY01 was dissolved in methanol combined with Celite (Merck, Germany), with the ratio of extract: Celite (1: 3) (w/w), then dried using rotary evaporator. The dried extract was fractionated using flash chromatography (Reveleris™, Buchi, Switzerland) and stationary phase column C-18 with water-acetonitrile as the mobile phase to obtain separate fractions. Flash chromatography was carried out based on the procedure manual for dry-loading samples [13]. The results of the active fraction obtained were then separated using the chromatotron method. From the results of the chromatotron separation, active isolates were obtained, and the results were observed using TLC densitometry and LC-MS spectroscopy methods.

### 2.3 Cytotoxic activity test of the active fraction on cancer cell culture

Cytotoxic activity test against MCF-7 breast cancer cells was carried out on all isolated fractions or active compounds. A total of 100 μL of MCF-7 cell culture suspension with a density of 1 × 10^5^ cells/mL and 100 μL of the test fraction or compound at various concentrations were put into a 96-well plate and incubated for 24 h at 37 °C. All treatments were performed in triplicate. After the incubation period was complete, the culture medium was discarded and replaced with 100 μL of the new complete medium and 10 μL of MTT solution [A 3-(4,5-dimethylthiazol-2-yl)-2,5-diphenyl tetrazolium bromide)] (Sigma, Aldrich). The well plates were incubated again for 4 h at 37 °C. After the incubation, 100 μL of 10 % SDS solution was added in 0.01 M HCl and incubated again for 24 h. Then the absorbance was read using an ELISA reader with a wavelength of 595 nm. The percentage of cell viability was calculated by comparing the absorbance value of the wells treated with the test compound with the absorbance of the negative control wells. The percentage of cell viability was then used to calculate the percentage of growth inhibition and to determine the concentration of the test compound that could inhibit cell growth up to 50 % (IC_50_). Cytotoxic activity test for the isolated active compound was also conducted against the Vero cell line. Statistical analysis using one-way ANOVA followed by posthoc test Tukey.

### 2.4 Structure elucidation of the active compound

#### 2.4.1 Spectrophotometry UV/Vis method

The active compound isolated from *Streptomyces* sp. GMY01 obtained was carefully weighed as much as 0.5 mg, then put in a 5 mL volumetric flask, then added with distilled water to the limit mark and obtained a concentration of 100 mg/mL. The solution was read for absorption at a wavelength between 200 and 800 nm using UV/Vis spectrophotometry to determine the maximum wavelength by looking at the highest and most stable absorption value.

#### 2.4.2 Infra-red spectroscopy (FT-IR) method

A total of 0.1 mg of the active compound sample was mixed with 20 mg of KBr and then pelleted. Measurements were made at wave numbers between 4000 and 400 cm^−1^. FT-IR spectrophotometer analysis was carried out to see the groups contained in the isolate of the active compound. The instrument used is the Buck Scientific Model 500 Infrared Spectrophotometer with a power supply of 100–240 V 50/60 Hz.

#### 2.4.3 Chromatography LC-MS method

The manufacture of mobile phase water: acetonitrile (3:1) is done by taking 300 mL of water and 100 mL of acetonitrile, then putting it in a 500 mL volumetric flask and adding distilled water to the limit, then filtering using a cellulose nitrate membrane filter. The isolated sample of the active compound was dissolved using methanol and injected into the LC-MS apparatus. The LC-MS apparatus was set in positive mode with a mass range between 50 and 1350 Da and the mobile phase flowed in a gradient for 25 min. The spectral results obtained show the fractional component of the groups contained in the isolate of the active compound.

#### 2.4.4 Spectroscopy NMR method

The ^1^H NMR and ^13^C NMR analysis tests were carried out with an NMR spectrometer (500 MHz; Jeol JNM-ECZ500R) in D_2_O solvent.

## 3 Result and discussion

In this study, the isolation of cytotoxic active compounds from *Actinomycetes* against the MCF-7 breast cancer cell line was carried out. Optimization of the *Streptomyce*s *sennicomposti* GMY01 fermentation was carried out to stimulate the optimal production of secondary metabolites.

To be able to produce secondary metabolites, *Streptomyces* must undergo a fermentation process using an appropriate medium. The result of the fermentation process is an extract containing a mixture of primary and secondary metabolites. The organic solvent extract of *S. sennicomposti* GMY01 obtained was then targeted for secondary metabolite profile analysis using TLC densitometry and LC-MS spectroscopy methods. The production of secondary metabolites from *Actinomycetes* is associated with several factors including nutrient media, pH, O_2_, temperature, and the addition of inducers or enzyme inhibitors [14,15]. Other environmental factors during fermentation, such as agitation speed can also affect the secondary metabolites production. In addition, the length of fermentation time used during fermentation also affects the amount of *Streptomyces* biomass formed and the antibiotic activity of the secondary metabolites produced (Narayana and Vijayalakshmi 2008; Kiranmayi et al., 2011; Sengupta et al., 2015).

In this study, the optimization of the culture media and different fermentation containers was carried out to optimize the growth of *S. sennicomposti* GMY01. Meanwhile, in optimizing the length of fermentation time, it was seen that starting on day 3, the *S. sennicomposti* GMY01 growth started to decline. Optimization to produce changes in the type and number of secondary metabolites was carried out by culturing *S. sennicomposti* GMY01 using variations of 2 different media (TSB and SNB), 2 different types of fermentation containers (regular Erlenmeyer and buffled Erlenmeyer), and 2 different fermentation durations (3 and 5 days) (see Table 1). It is known that carbon sources such as fructose, glycerol, starch, and sucrose in culture media can lead to increased production of antibiotics by *Streptomyces* spp [16,17]. while the decrease in secondary metabolite production can be caused by the presence of glucose, ammonia, and phosphate [16,18]. The color change of the extract produced by *S. sennicomposti* GMY01 is an early indicator that supports the changes in nutrients in the fermentation media can cause changes in the secondary metabolites produced. The color change was seen when *S. sennicomposti* GMY01 was cultured using SNB media which showed a more intense color than when cultured using TSB media.

**Table 1:** The best IC_50_ value of the extract optimization of *Streptomyces sennicomposti* GMY01 on MCF-7 breast cancer cell line.

No. Sample	Medium	Fermentor	Duration of Fermentation	Extract Part	Solvent	IC_50_ (μg/mL)
1	SNB	*Buffled*	3 days	*Pellet*	Methanol	858
2	TSB	*Erlenmeyer*	3 days	*Pellet*	Methanol	1308
3	SNB	*Buffled*	5 days	*Pellet*	Methanol	245
4	SNB	*Erlenmeyer*	5 days	Supernatant	Ethyl acetate	89
5	SNB	*Buffled*	3 days	Supernatant	Ethyl acetate	893
6	TSB	*Buffled*	3 days	Supernatant	Ethyl acetate	421
7	TSB	*Buffled*	5 days	Supernatant	Ethyl acetate	555
8	SNB	*Erlenmeyer*	5 days	*Pellet*	Methanol	78

The methanol extract of *Streptomyces* sp. GMY01 had almost the same metabolite profile, both cultured using TSB and SNB media. However, based on the resulting cytotoxic activity, the bacteria *S. sennicomposti* GMY01 cultured with SNB media had the best cytotoxic activity compared to other extracts. This indicates that although they have similar metabolite profiles, there are still differences in the metabolite profiles produced and the cytotoxic activity obtained. Differences in culture media can cause different profiles of secondary metabolites produced by isolates of *S. sennicomposti* GMY01. This study is in accordance with several studies that have been carried out previously, which have found differences in the characteristics of the secondary metabolite profile of 1 isolate cultured using 10 mediums and analyzed using HPLC chromatogram data [19]. In addition to using secondary metabolite profile analysis, biological activity data in this case cytotoxic activity can also be used to obtain unique compounds that are predicted to influence these activities. From the results of the study, it was found that culture media using SNB showed stronger cytotoxic activity on MCF-7 cells than TSB media. SNB media has been widely used in research for the fermentation process of a bacterium. Previous research stated that SNB media was used for the fermentation process of 5 types of bacteria isolated from the rhizosphere of the *Ficus carica* L. plant and an antibacterial test was carried out on ethyl acetate extract from bacterial pellets [20]. From the results of the study, it was found that there was 1 spot in the extract which had an effect as an antibiotic. In addition, the *Bacillus* sp. bacteria which was cultured in SNB media produced sericin compounds that were proven to have cytotoxic activity in several cancer cell cultures, which were Caco-2, MCF-7, and A549 cells [21]. In a previous study, an extract of *S. sennicomposti* GMY01 which was fermented using SNB medium showed anticancer activity on human cancer cell line A549 [12].

**Fig. 1:**
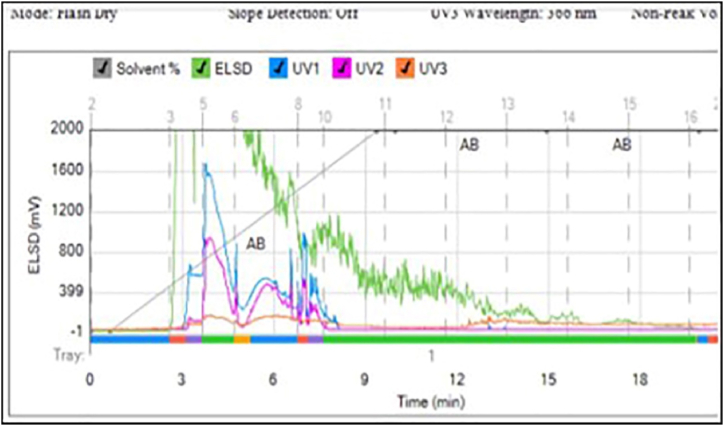
A fractionation results of methanol extract from *Streptomyces sennicomposti* GMY01 using Flash Chromatography Method.

Based on the result of the extract separation using the flash chromatography method obtained 9 fractions (Fig. 1). Then the fractionation extract was tested for cytotoxic activity to choose the most potent cytotoxicity activity of the fraction.

**Fig. 2:**
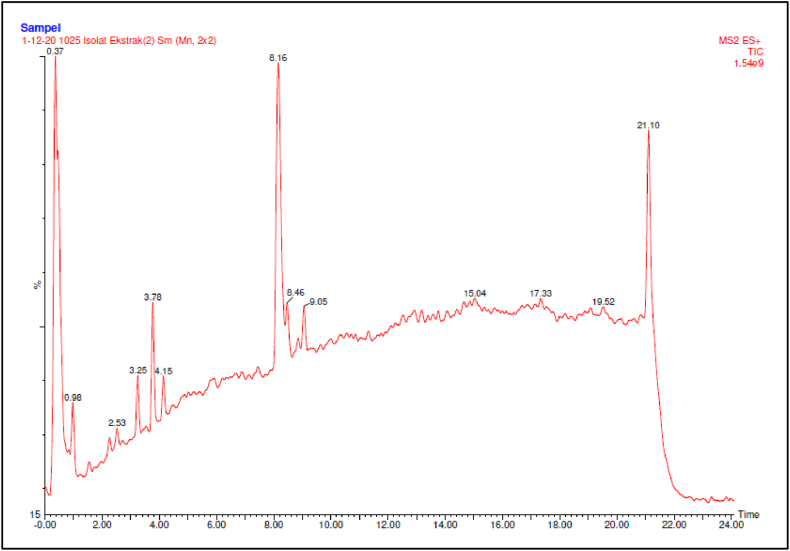
Active compound of *Streptomyces sennicomposti* GMY01 analysis using LC-MS spectroscopy method.

Based on the result of active compound analysis using the LC-MS spectroscopy method showed that 1 dominant peak at RT: 8.16 min (Fig. 2). From the results of the LC method analysis, it is suspected that the active compound responsible for the cytotoxic effect is the dominant peak at a retention time of 8.16 min. From the results of the LC method analysis, fractions were then observed as active compounds at the dominant peak of RT: 8.16 min using the MS method. From the results of MS analysis, it is known that the most active compound This dominant has a BM value of 334.65 Da. This matter can be the BM of the active compound or a fraction of compounds contained in the isolate.

**Fig. 3:**
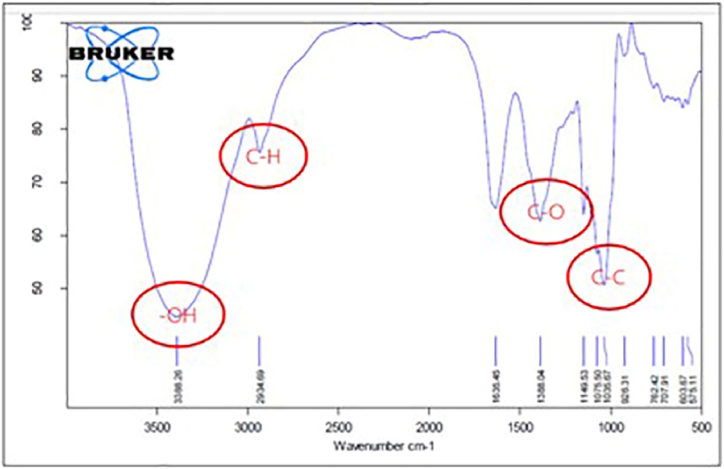
Active compound of *Streptomyces sennicomposti* GMY01 analysis using FT-IR spectrophotometry method.

Based on the result of active compound analysis using the FT-IR spectroscopy method showed several functional groups in the peak (Fig. 3). From the results of FT-IR spectroscopy analysis, there is a peak at a wavelength of 1035–1149 cm-1 with a stretching vibration type which is a typical peak for the alkane group. Furthermore, there is also a peak at wavelength 1388 cm-1 which leads to C–O. The peak is at a wavelength of 1635 cm-1 with a bending vibration type which is the C=O group. The wavelength of 2934 cm-1 is typical of the C–H group and the wavelength of 3388 cm-1 is typical of the –OH group.

Based on the results of the isolation of the active compounds of the selected extracts, it was known that the isolates of the active compounds were in the form of mannotriose compounds. This is shown from the data analysis of ^1^H NMR and ^13^C NMR which leads to the structure of these compounds. In addition, the LC-MS/MS analysis data showed that the active fraction of the methanol extract of *S. sennicomposti* GMY01 also contains mannotriose compounds [10]. The results of the 1H NMR analysis show that there is a typical peak of hydrogen atoms in the saccharide ring at a chemical shift of 3.4–4.0 ppm.

**Fig. 4:**
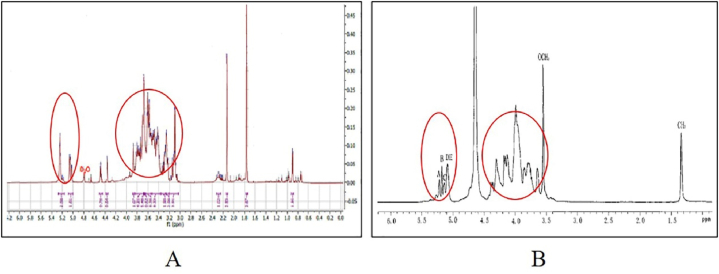
The ^1^H NMR spectral analysis profile of active compound isolates from *Streptomyces sennicomposti* GMY01 with solvent D_2_0 (A), isolate the fungal compound *Phellinus igniarius* with solvent D_2_0 (B) [22].

In addition, there is also a typical peak of anomeric hydrogen atoms at a chemical shift of 5.0–5.4 ppm. This is in accordance with the previous study conducted in by Yang et al. [22], who conducted an analysis of isolate compounds in the form of polysaccharides obtained from the fungus *Phellinus igniarius* (Fig. 4).

**Fig. 5:**
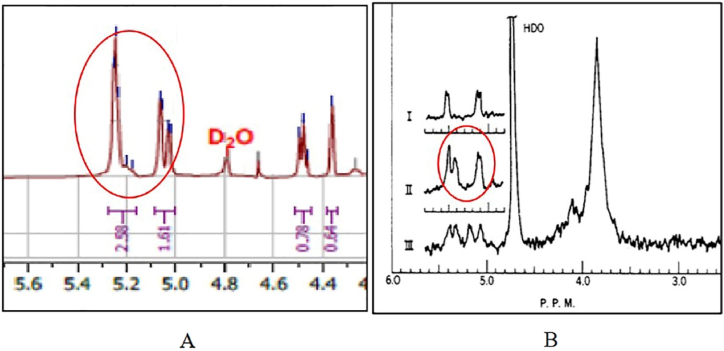
Analysis of anomeric hydrogen peaks in ^1^H NMR spectra of active compound isolates from *Streptomyces sennicomposti* GMY01 with solvent D_2_0 (A), isolate compounds in the fungus *Phellinus igniarius* with solvent D_2_0 [23] (B).

The typical peak of hydrogen atoms at a chemical shift of 3.4–4.0 ppm is one of the main characteristics of the presence of saccharide groups in these compounds. Where to determine the number of sugar groups that make up the compound, can be seen from the peak profile of the anomeric hydrogen atoms that appear on the chemical shift between 5.0 and 6.0 ppm. The number of anomeric hydrogen peaks indicates the number of sugar groups that make up the isolated compound. From the observation, it is known that the peak profile of anomeric hydrogen in the isolate of the active compound of the bacterium *Streptomyces* sp. GMY01 contains 3 hydrogen peaks, so it is suspected that the isolate of the active compound has 3 constituent sugar groups. This is also consistent with previous research about the preparation and analysis of mannobiose, mannotriose, and mannotetraose compounds from the yeast *Saccharomyces cerevisiae* [23] (Fig. 5). From the comparison results of the anomeric hydrogen peaks contained in the isolates of the active compound of the bacterium *S. sennicomposti* GMY01 has a close resemblance to the peak of the mannotriose compound in the isolated compound from the yeast *Saccharomyces cerevisiae*.

**Fig. 6:**
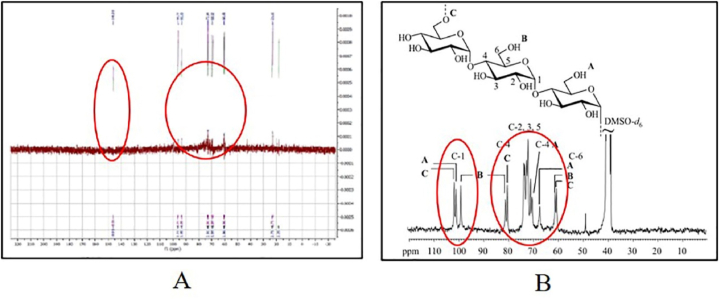
Analysis profile on ^13^C NMR spectra of active compound isolates from *Streptomyces sennicomposti* GMY01 with solvent D_2_0 (A), isolate compound on *Aurerobacidium pullulan* with solvent DMS0-d6 [32] (B).

Based on the ^13^C NMR analysis data, it is known that the active compound isolates of *S. sennicomposti* GMY01 contain a typical carbon atom spectrum for sugar groups in the chemical shift between 60 and 100 ppm. In addition, there is also a typical peak for the anomeric carbon atom in the chemical shift between 140 and 150 ppm (Fig. 6).

Until now, there is still limited research on candidates for anticancer drugs based on polar carbohydrates. Previous studies have shown that antitumor activity can be carried out by increasing solubility by adding hydrophilic functional groups such as sugars (glucose, galactose, and mannose) [24]. In recent years, several carbohydrate compounds have been developed for various medicinal applications ranging from compounds with antibiotic, antiviral, or fungicidal activity to anticancer compounds [25]. Some of them are polysaccharide anticancer compounds (CPP) isolated from *Cyclocarya paliurus* which were tested against the inhibition of HeLa gastric cell human carcinoma [26]. There are 3 novel polysaccharide acids (PRM1, PRM3, and PRM5) isolated from the roots of *Rhynchosia minima* which have anticancer effects on lung cancer A549 and liver cancer cells HepG2 [27]; The original and modified fucoidan fractions that have galactose residues also have anticancer activity against DLD-1 and HCT-116 colorectal carcinoma cells [28]. Galactose is a water-soluble carbohydrate that has high hydrophilic properties. In a previous study, it was shown that the compound paclitaxel (taxol), a diterpenoid originally isolated from the bark of the Taxus brevifolia tree, is currently used in the treatment of various types of cancer but has limited water solubility, which hinders its use as an anticancer agent. Attempts at synthesizing the galactose-bound taxoid analog paclitaxel (10-a-GAG-DT) proved to be superior to all other taxoids in water solubility and antitumor activity [24].

**Fig. 7:**
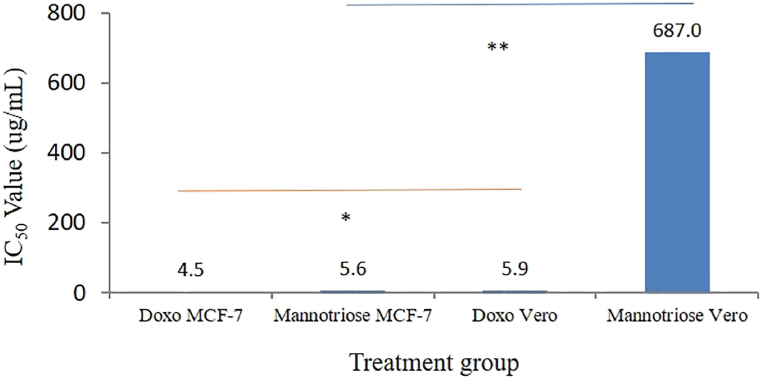
Comparison of the cytotoxic value of doxorubicin and isolates of the active compound mannotriose in MCF-7 cancer cells and Vero normal cells. All treatments were performed in triplicate. The * sign indicates the data is not significantly different, **indicates the data is significantly different. Statistical analysis using one-way ANOVA followed by posthoc test Tukey.

From the results of the cytotoxic test, the isolated compound suspected to be a manntriose compound was known to have an IC_50_ value of MCF-7 cancer cells of 5.6 μg/mL, while the drug doxorubicin had an IC_50_ value of 4.5 μg/mL. Based on research in the previous study showed that the IC_50_ value of a compound fraction or isolate with an IC_50_ value of less than 10 μg/mL is included in the category of the very strong cytotoxic agent [29]. So, it can be concluded that both doxorubicin and mannotriose compound isolates are categorized as very strong cytotoxic agents against MCF-7 breast cancer cells. In the selectivity test of the active compound isolate, mannotriose was analyzed on normal Vero cells. From the results of the cytotoxic test on normal Vero cells of the active isolate compound mannotriose and doxorubicin, the IC_50_ values were 687 and 5.9 μg/mL (Fig. 7). Thus, based on these data, the selectivity index (SI) of the active compound isolate mannotriose is 122 times, while for doxorubicin it is 0.8 times. The small SI data for the drug doxorubicin are also in line with those of Wang et al. (2017). From these results, it is known that the drug doxorubicin has an IC_50_ value of 1.0 μM in MCF-7 cancer cells, and 0.4 μM in normal Vero cells, so it has an SI value of 0.4 times. Based on research from previous study, the SI value of a compound fraction or isolate with an SI value of more than 3 times is included in the category of cytotoxic agents with good selectivity [29]. So based on this statement, it can be concluded that the active isolate compound mannotriose has a very good SI value, while the drug doxorubicin has a poor SI value. This very good SI value illustrates that the active isolate compound mannotriose has a relatively high degree of safety in normal cells compared to doxorubicin.

It has been reported mechanism of carbohydrate-based compounds is anticancer. Many studies have reported that the bioactivity of polysaccharides is largely related to their physicochemical properties, including polysaccharide content, molecular weight, and type of sugar. Other studies have also reported that the anticancer activity of some polysaccharides is associated with free radical stabilizing activity, specific conformation and affinity for cell surface receptors, and antioxidant enzyme activity [26]. Other polysaccharide anticancer compounds, fucoidans derivatives have biological activities that depend on different structural characteristics such as monosaccharide composition, molecular weight, presence of branches, and content of sulfate and acetyl groups [30]. In vitro antitumor studies of the compound prumicin also showed inhibition of protein synthesis and DNA synthesis in HeLa cancer cell cultures [31].

## 4 Conclusion

The active compounds of mannotriose were isolated from a methanol extract of *Streptomyces sennicomposti* GMY01 which has strong cytotoxic activity against MCF-7 breast cancer cells line with IC_50_ value of 5.6 μg/mL and has high selectivity index on Vero normal cells line (SI = 122).

## CRediT authorship contribution statement

**Rifki Febriansah:** Conceptualization, Formal analysis, Investigation, Writing – original draft, Writing – review & editing, Visualization. **Triana Hertiani:** Conceptualization, Data curation, Resources, Supervision. **Jaka Widada:** Conceptualization, Methodology, Resources, Supervision. **Muhammad Taher:** Conceptualization, Supervision, Validation. **Ema Damayanti:** Conceptualization, Formal analysis, Methodology, Resources, Writing – original draft, Writing – review & editing. **Mustofa Mustofa:** Conceptualization, Data curation, Funding acquisition, Project administration, Supervision, Writing – review & editing.

## Declaration of competing interest

The authors declare that they have no known competing financial interests or personal relationships that could have appeared to influence the work reported in this paper.
